# Redox-Responsive Hydrogels for Tunable and “On-Demand” Release of Biomacromolecules

**DOI:** 10.1021/acs.bioconjchem.2c00094

**Published:** 2022-04-21

**Authors:** Ruveyda Kilic Boz, Duygu Aydin, Salli Kocak, Bianka Golba, Rana Sanyal, Amitav Sanyal

**Affiliations:** † Department of Chemistry, Bogazici University, Istanbul 34342, Turkey; ‡ Center for Life Sciences and Technologies, Bogazici University, Istanbul 34342, Turkey

## Abstract

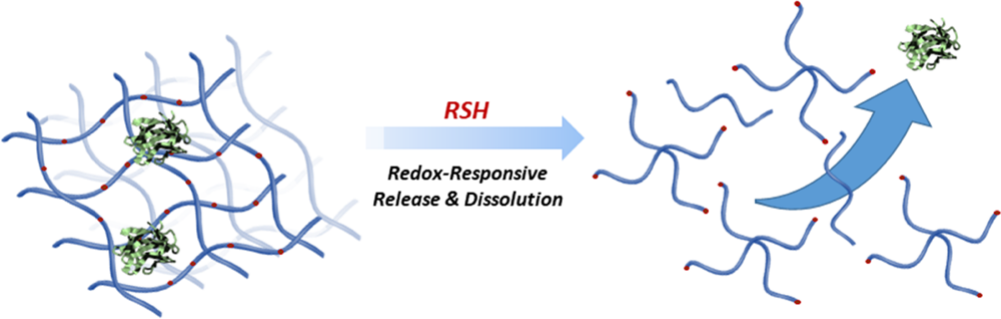

In recent years, stimuli-responsive degradation has emerged as a desirable design criterion for functional hydrogels to tune the release of encapsulated payload as well as ensure degradation of the gel upon completion of its function. Herein, redox-responsive hydrogels with a well-defined network structure were obtained using a highly efficient thiol-disulfide exchange reaction. In particular, gelation occurred upon combining thiol-terminated tetra-arm polyethylene glycol (PEG) polymers with linear telechelic PEG-based polymers containing pyridyl disulfide units at their chain ends. Rapid gelation proceeds with good conversions (>85%) to yield macroporous hydrogels possessing high water uptake. Furthermore, due to the presence of the disulfide linkages, the thus-obtained hydrogels can self-heal. The obtained hydrogels undergo complete degradation when exposed to environments rich in thiol-containing agents such as dithiothreitol (DTT) and L-glutathione (GSH). Also, the release profile of encapsulated protein, namely, bovine serum albumin, can be tuned by varying the molecular weight of the polymeric precursors. Additionally, it was demonstrated that complete dissolution of the hydrogel to rapidly release the encapsulated protein occurs upon treating these hydrogels with DTT. Cytotoxicity evaluation of the hydrogels and their degradation products indicated the benign nature of these hydrogels. Additionally, the cytocompatible nature of these materials was also evident from a live/dead cell viability assay. One can envision that the facile fabrication and their ability to degrade on-demand and release their payload will make these benign polymeric scaffolds attractive for various biomedical applications.

## Introduction

In recent years, hydrogels have emerged as an attractive scaffold for a variety of biomedical applications such as diagnostics, delivery of therapeutic agents, and tissue engineering.^1−4^ Due to their hydrophilic network structure, which provides a benign environment for delicate biomolecules, they have been extensively investigated for localized, prolonged release of biopharmaceuticals such as proteins and peptides. Also, the ability to design hydrogels to resemble the natural tissue in terms of chemical and mechanical properties makes them suitable scaffolds for tissue engineering.^5^ Traditionally, release of encapsulated molecules was achieved through diffusion and/or passive degradation of hydrogels. In recent years, introduction of stimuli-responsive elements into the hydrogel matrix has gathered interest. Such stimuli-responsiveness can be used to trigger the release of therapeutic agents, provide modulation of mechanical strength of the matrix during cell proliferation, or just provide a method to dissolve and remove the hydrogel matrix after its application.^6−14^ The last aspect is quite important when hydrogels are used as bandages during healing of infected wounds or during recovery from burns.^15,16^ The removal of hydrogels in such cases can inflict pain and disturb the healed tissue if not removed in a mild manner. Thus, facile access to on-demand degradable and/or easily dissolvable hydrogels can find many applications.

Among various approaches available to introduce “on-demand” degradable linkages into polymeric materials, utilization of disulfide-based redox-sensitive linkages has gathered a lot of interest in recent years due to their potential applications in drug delivery.^17−21^ In most cases, such linkages have been introduced during the formation of hydrogels by using disulfide-containing or cyclic thiosulfinate-based crosslinkers or macromonomers.^22−24^ While most of the work in this area has focused on free-radical polymerization-based hydrogel formation in the presence of crosslinkers like disulfide-containing bis-methacrylamides, the other common approach has been the use of disulfide-based crosslinkers to achieve post-polymerization interchain crosslinking. In either cases, the nature of the crosslinking is random and does not warrant homogeneous network formation. In recent years, there is increasing interest in obtaining a more homogeneous and well-defined network structure since such structures have enhanced swelling and mechanical properties, compared to randomly crosslinked hydrogels.^25^ In this regard, reactions with high efficiency, such as click reactions, have attracted interest in both fabrication and functionalization of hydrogels.^26,27^ One can envision that compared to random crosslinking, fabrication of redox-responsive networks with near well-defined chain connectivity will provide a handle of control over the release characteristics of encapsulated macromolecular agents like therapeutic proteins. A general approach to fabricate redox-responsive disulfide-containing polymeric materials that has attracted widespread interest in recent years involves the utilization of the thiol-disulfide exchange reaction.^28^ In particular, activated disulfide groups such as the pyridyl-disulfide have been extensively utilized in installation of disulfide linkages along the side chains,^29,30^ backbone,^31^ chain ends,^32^ chain junctions,^33^ and random crosslinking in nanogels^34^ and hydrogels.^35^ The exchange reaction is quite fast and proceeds with high efficiency under mild reaction conditions. Thus, we envisioned that this efficient reaction can be utilized for the fabrication of near well-defined hydrogels with precisely located redox-responsive cleavable junctions.

**Scheme 1:**
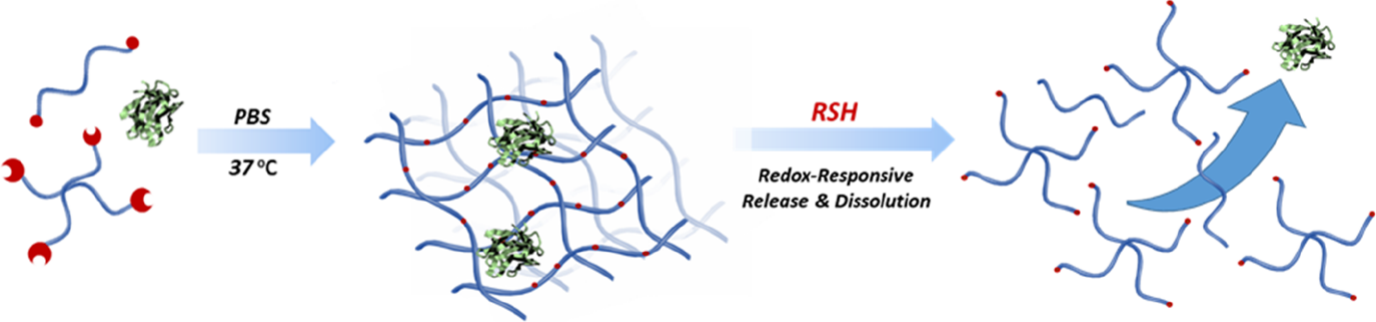
Schematic Illustration of Fabrication and Degradation of Redox-Responsive Hydrogels

Herein, we propose facile fabrication of a disulfide-containing hydrogel using the thiol-disulfide exchange reaction to obtain a modular platform for encapsulation of biomacromolecules and their sustained and on-demand release (Scheme 1). In particular, linear poly(ethylene glycol) (PEG) polymers were functionalized at their chain ends with pyridyl disulfide groups, which upon mixing with tetra-arm thiol-containing PEG yielded hydrogels within minutes in PBS (pH 7.4) media at 37 °C, with high conversions. The obtained hydrogels were characterized for their swelling, morphology, and rheological properties. The change in their rheological properties and ability to self-heal were investigated using rheological measurements. Encapsulation of a model biomolecule, bovine serum albumin (BSA), was investigated to probe the passive and active diffusion in the presence of a thiol-containing reducing agent. Additionally, biocompatibility and cytotoxicity of these hydrogels and their degradation products were investigated on fibroblast cell lines to show that these soft materials are suitable for biological applications.

## Results and Discussion

### Synthesis of Hydrogel Precursors

**Figure 1:**
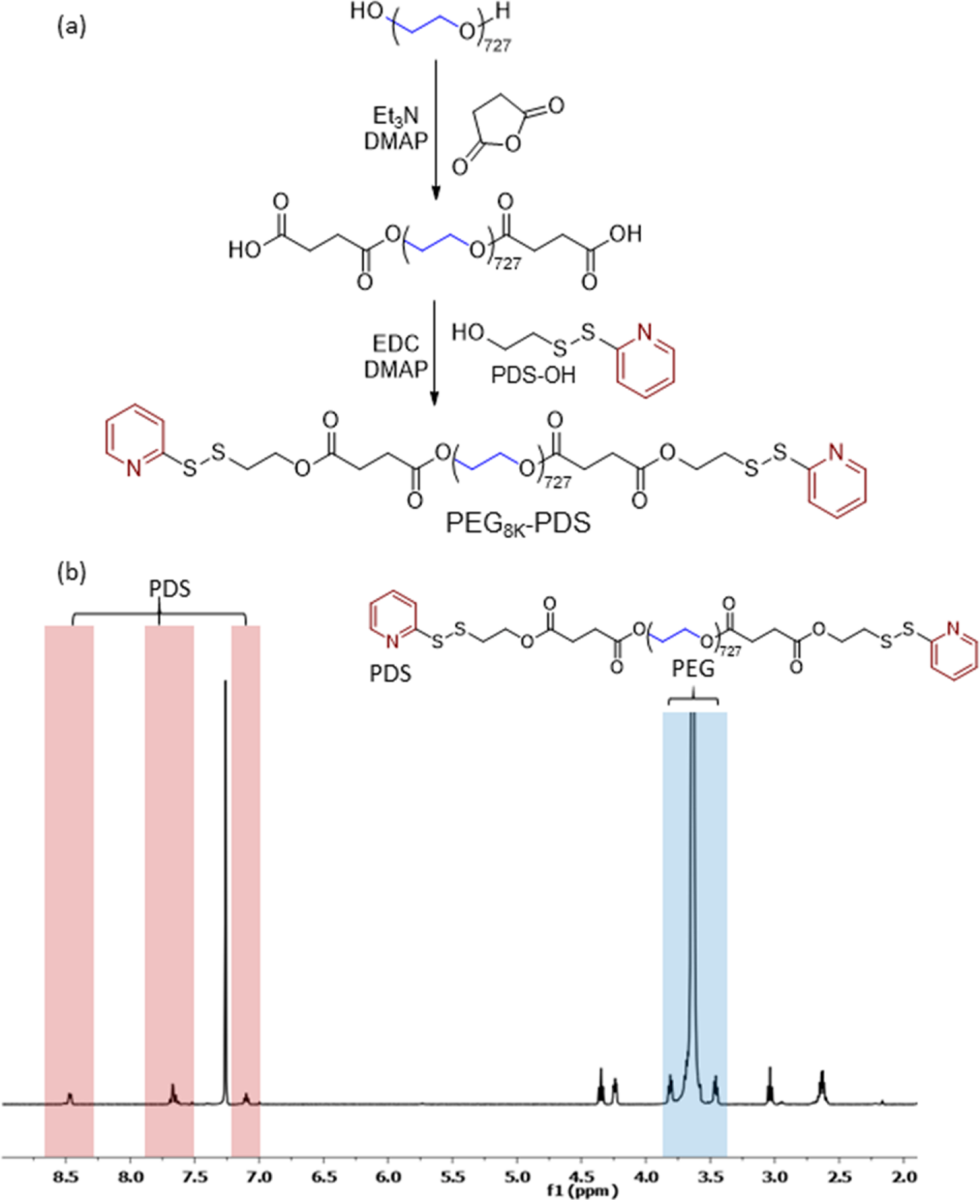
(a) Synthesis of a disulfide-containing PEG8K-PDS macromonomer and (b) ^1^H NMR spectrum in CDCl_3_.

Redox-responsive hydrogels were obtained by mixing linear PEG polymers containing reactive PDS groups at their chain ends with tetra-arm PEG-thiol macromonomers. PDS-containing linear PEG polymers with two different molecular weights (*M*_n_: 2 and 8 kDa), hereafter denoted as PEG_2K_-PDS and PEG_8K_-PDS, respectively, were utilized to fabricate hydrogels with different physical and mechanical properties. The PEG-PDS macromonomers were prepared from commercially available linear PEG polymers (Figure 1). First, the hydroxyl groups at chain ends were converted to carboxylic acids through reaction with succinic anhydride in the presence of organobase catalysts Et_3_N and DMAP.^36^ Subsequently, the thiol-reactive PDS end groups were installed through conjugation of pyridyl disulfide alcohol (PDS-OH), which was prepared according to a previously reported procedure.^37^ Quantitative chain-end functionalization of linear PEGs with the PDS moiety was confirmed from the peak integration values in ^1^H NMR spectra (Figures S1–S4) as well as from the expected complete disappearance of carbon resonances at 72.8 ppm belonging to the carbon atom bearing the terminal hydroxyl group, as deduced from ^13^C NMR spectra (Figures S5 and S6).^38^ Also, as expected no significant change in molecular weight of polymers was observed upon end-group modifications (Figure S7), and the emergence of the carbonyl band stretching peaks was evident (Figure S8). The tetra-arm PEG_10K_-SH was prepared according to a literature procedure,^39^ by acid-catalyzed esterification of tetra-arm PEG_10K_-OH with mercaptopropionic acid. The quantitative presence of chain-end thiol functional groups was confirmed through treatment with Ellman’s reagent, according to a previously used protocol.^40^

### Preparation and Characterization of Hydrogels

**Figure 2:**
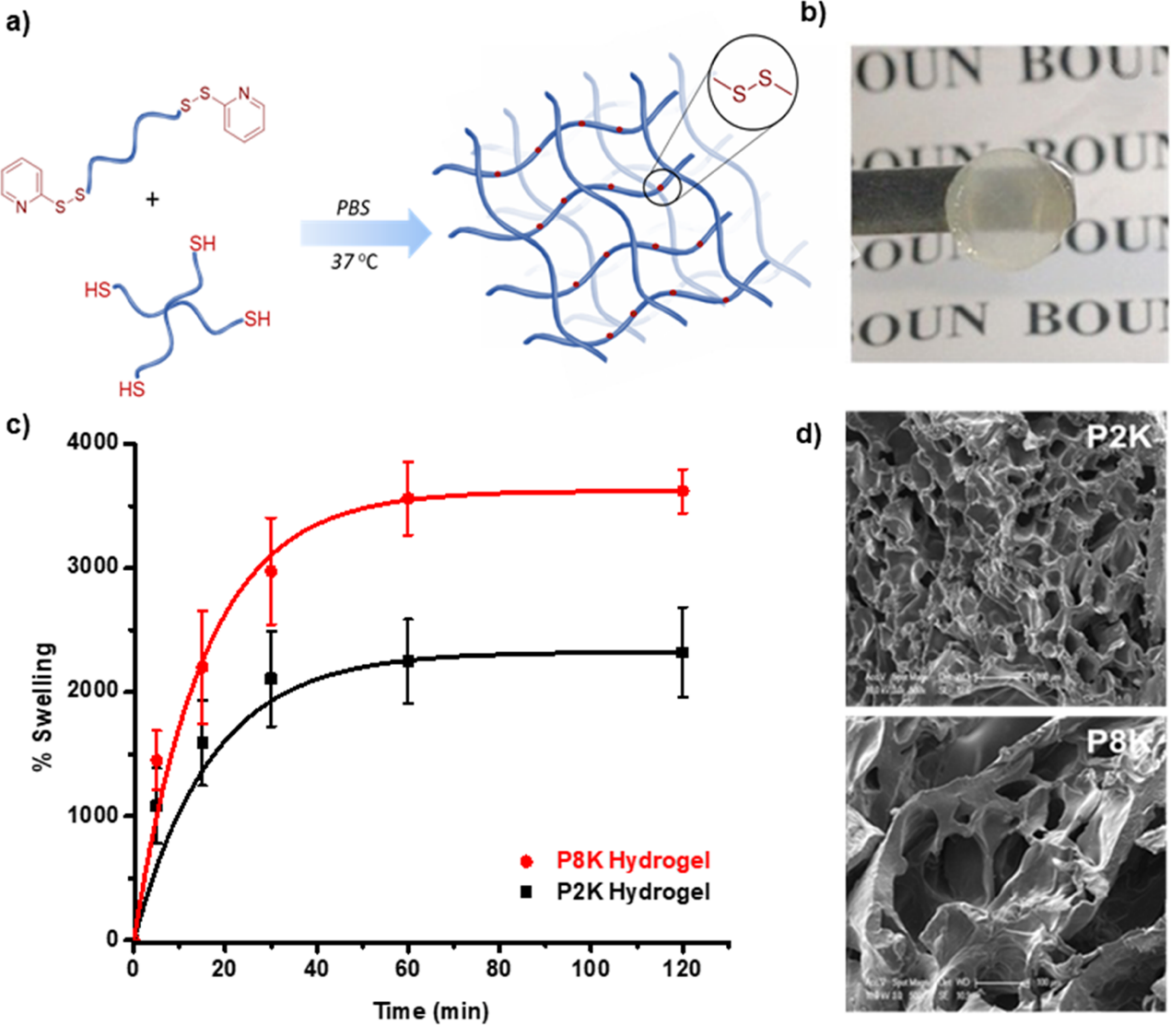
(a) Synthesis of hydrogels, (b) representative photograph of a hydrogel, (c) water uptake swelling profiles, and (d) SEM micrographs of hydrogels fabricated using PEG2K (black) and PEG8K (red) linear PEG-based precursors.

Hydrogel formation was achieved by mixing equimolar aqueous solutions 33% (w/v) of PEG-PDS and tetra-arm PEG_10k_-SH polymers in PBS buffer (pH 7.4) at 37 °C (Figure 2a). Semi-transparent (in the wet state) hydrogels were obtained with yields greater than 85%, as determined gravimetrically. The thiol and pyridyl-disulfide end group consumptions were in the ranges of 97–99% for P2K and 95% for P8K hydrogels, respectively. To investigate the swelling behaviors of the hydrogels, freshly prepared gels were lyophilized and immersed in aqueous media (Figure 2b). It was observed that the water uptake increased with the increase in polymer chain length. The equilibrium swelling was up to 3600% for the P8K gel and 2300% for the P2K gel (Figure 2c). The porous morphology of the hydrogels was evident from the scanning electron microscopy (SEM) images of the freeze-dried hydrogels (Figure 2d).

### Rheological Characterization and Self-Healing Behavior of Hydrogels

**Figure 3:**
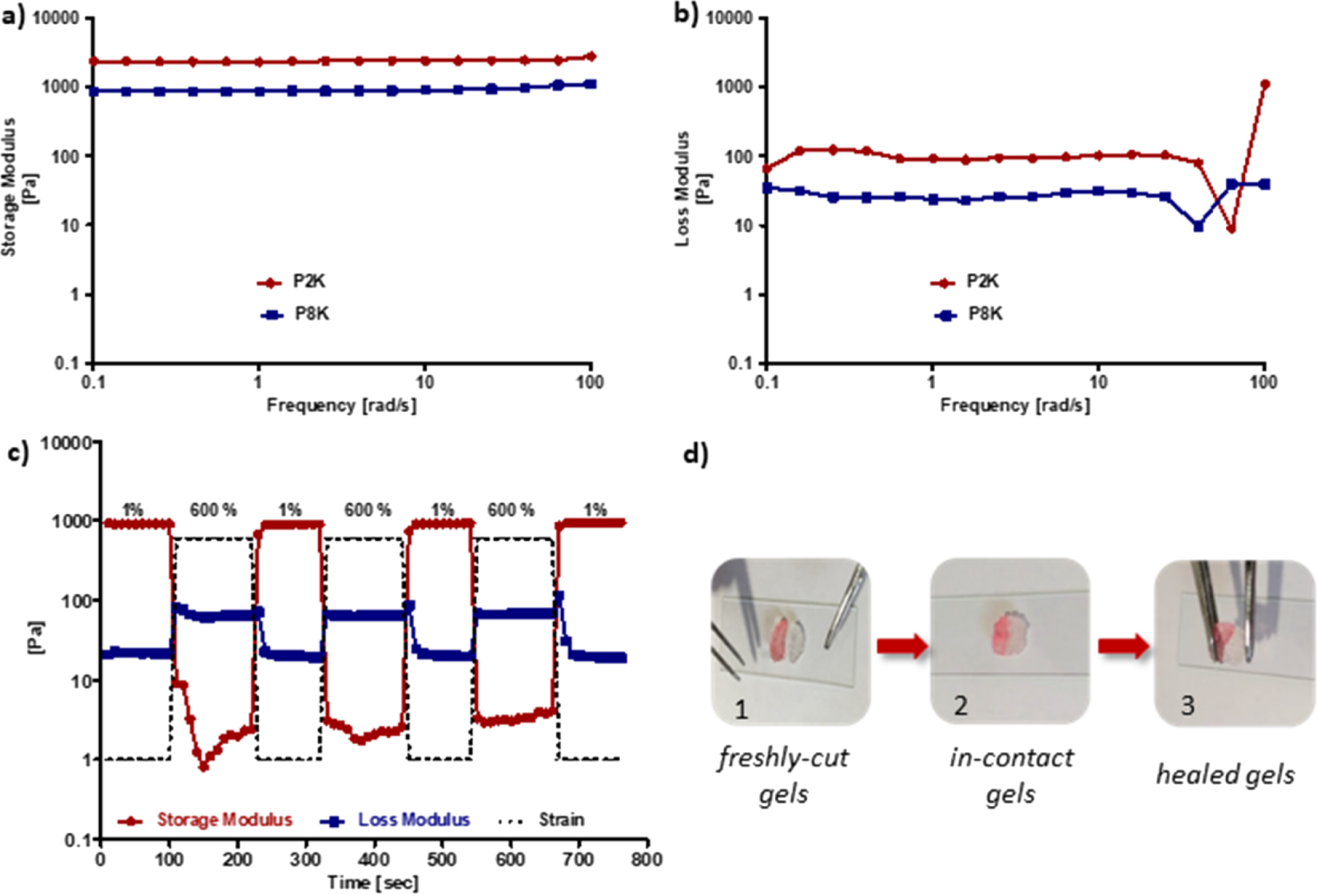
Frequency sweep tests showing (a) storage and (b) loss moduli of P2K and P8K hydrogels, (c) rheological self-healing test for the P8K hydrogel showing storage modulus (*G*′) and loss modulus (*G*″) at alternating strains of 1 and 600% (three cycles), and (d) macroscopic self-recovery images of the P8K hydrogel: (d-1) freshly fabricated hydrogels (with and without food dye) and (d-2,3) freshly cut gel pieces placed in contact and their healing to a single piece.

Fabricated hydrogels were subjected to rheological tests to understand their viscoelastic properties. Frequency sweep tests across the angular frequency range of 0.1–100 Hz using a strain value of 1% were carried out to investigate the stiffness of hydrogels (Figure 3a,b). Both P2K and P8K hydrogels were mechanically stable and robust and showed gel-like properties since the storage modulus was higher compared to the loss modulus. The P2K hydrogel possessed a higher *G*′ (storage modulus) compared to the P8K hydrogel as expected due to the shorter chain of the polymers involved in crosslinking. In recent years, interest in self-healing of hydrogels has increased since they can improve their lifetime and performance; hence, we also investigated this aspect for our hydrogels.^41−43^ To understand the self-healing behavior of the P8k hydrogel, as a representative, first the strain-dependent deformation of the hydrogel was investigated by varying the strain between 0.01 and 1000% (Figure S9). Then, for the assessment of self-healing, extreme (600%) and mild (1%) strains were applied onto the P8K hydrogel in alternating cycles. Under 600% strain, the storage modulus (*G*′) decreased drastically from almost 1000 Pa to 1 Pa and became lower than the loss modulus (*G*″) due to the rupture and deformation in the gel structure. Upon reducing the strain to 1%, an instant recovery of *G*′ to its original value was observed (Figure 3c). This quick recovery can be attributed to the dynamic nature of the disulfide bonds. Thus, the obtained hydrogel possessed the ability to recover its mechanical strength without any external intervention after it is damaged, as long as they remain in contact. As a control experiment, the P8K hydrogel was incubated for 2 h in PBS containing ethyl-maleimide and subjected to the self-healing test with the same conditions. Ethyl-maleimide was used as a capping agent for any thiol-containing groups formed upon rupture of the disulfide linkages. Upon repeated strain cycles, the ethyl maleimide-soaked hydrogel exhibited poor recovery, and a decrease in its mechanical integrity was evident from loss of storage modulus after each strain cycle (Figure S10).

The self-healing property of the PEG-based disulfide-containing hydrogels was further investigated visually to ascertain the ability of hydrogels to merge into a singular unit when freshly cut pieces of hydrogels were brought in contact with each other. Two separate samples of the P8K polymer-based hydrogel were prepared, and one of them was imparted with a pink color using a food dye. Both hydrogel samples were cut into two equal semicircular pieces by a surgical blade. The two dissimilar halves that were kept in contact allowed to self-heal for about 5 min. Then, the merged gel was stretched with the help of a tweezer to confirm that the two halves were robustly joined through self-healing (Figure 3d).

### Degradation of Redox-Responsive Hydrogels

**Figure 4:**
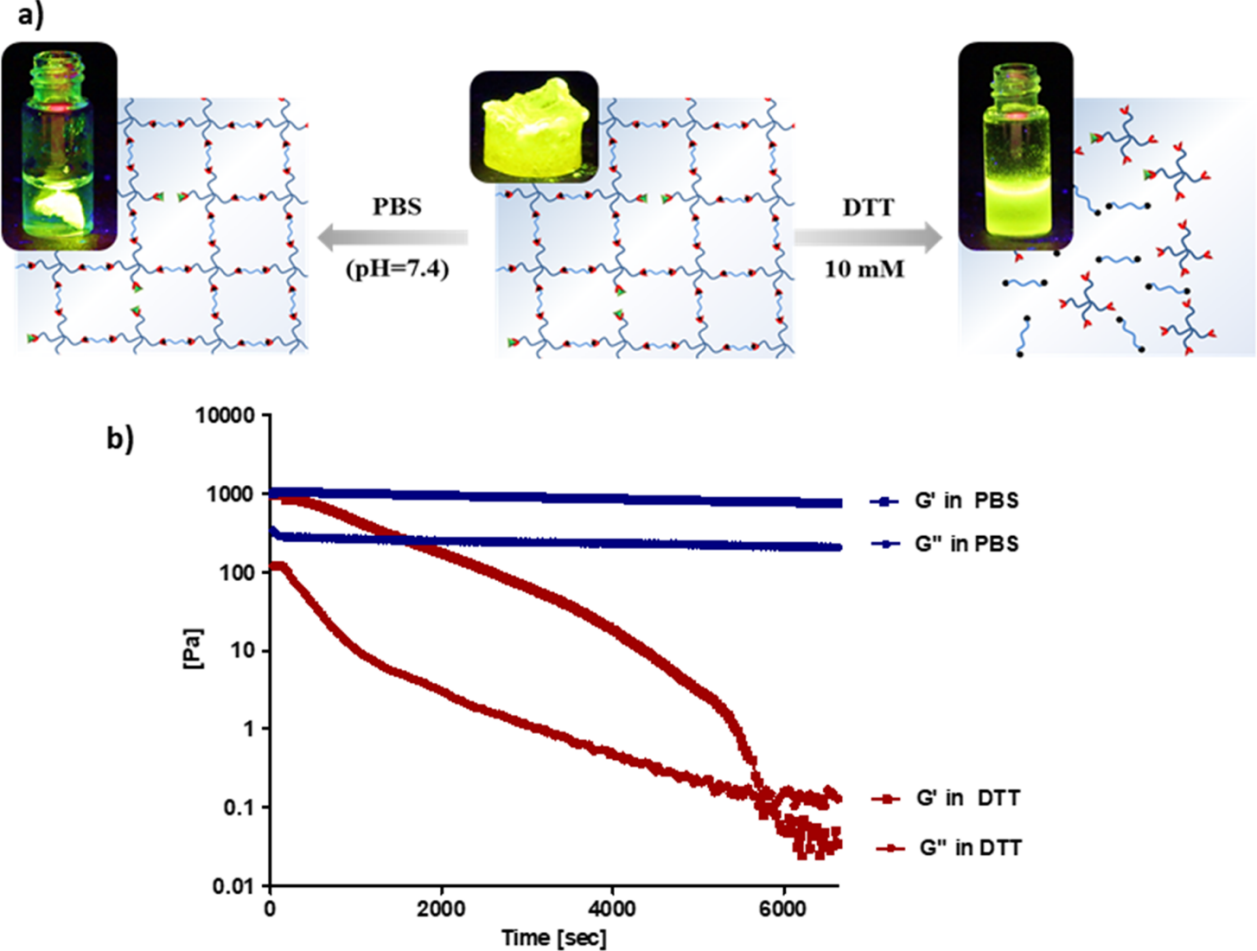
(a) Visual degradation of the dye-conjugated hydrogel (P8K) in PBS and DTT (10 mM) and (b) rheological plots of storage and loss modulus of the hydrogel (P8K) incubated in PBS (blue plot) and DTT (10 mM) (red plot) at 37 °C.

Disulfide-containing hydrogels are expected to be degradable in the presence of a reducing agent such as dithiothreitol (DTT). To this end, fluorescent dye-conjugated disulfide-containing hydrogels were prepared by treatment of the residual trace amount of thiol groups within hydrogels with fluorescein-maleimide to visualize the redox-responsive degradation. Dye-conjugated gels were treated with PBS and DTT-containing PBS (10 mM) buffer, respectively. A piece of dye-modified hydrogel was immersed in PBS, and it was observed to remain intact and not give any distinct fluorescence to the solution (Figure 4a, left). However, a homogeneous bright green solution was observed under UV illumination (365 nm) in the DTT-containing vial due to the disintegration of the dye-incorporated gel (Figure 4a, right). A similar trend was observed when the hydrogels were incubated in a solution of the endogenous reducing agent GSH (5 mM) and PBS buffer. While complete dissolution of the hydrogel was observed in the reducing environment, the sample immersed in PBS buffer remained intact (Figure S11). Additionally, redox-responsive degradation was investigated in the DTT-containing reductive environment using a rheometer at 37 °C, using time sweep tests for the P8K gel. It was observed that the *G*′ of the hydrogel gradually decreased and a crossover was observed in the DTT-containing medium, indicating a damaged network and the loss of gel-like properties upon cleavage of disulfide bonds. Also, as expected, the *G*′ and *G*″ values did not change over time for the hydrogel samples incubated in PBS (7.4) (Figure 4b).

### Encapsulation and Release of Protein from Redox-Responsive Hydrogels

**Figure 5:**
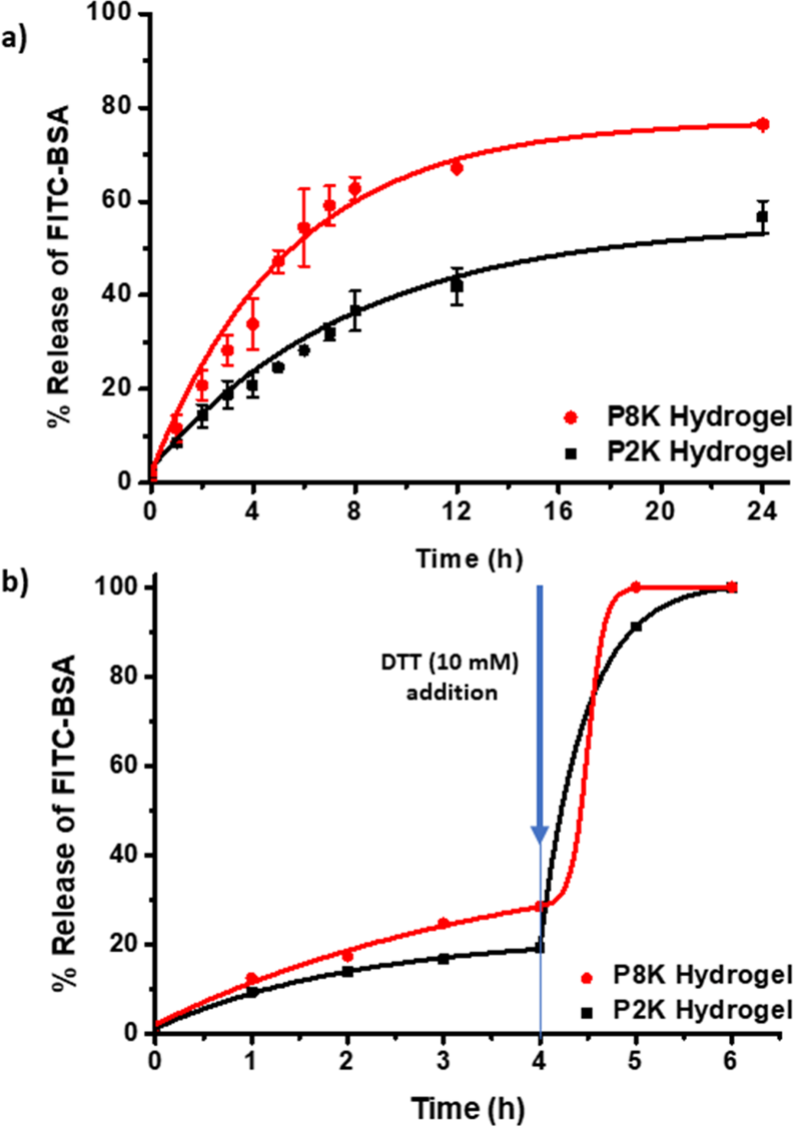
(a) Passive release profiles of FITC-BSA from the hydrogel fabricated using PEG8K and PEG2K and (b) forced release of FITC-BSA upon addition of DTT (10 mM) after 4 h of passive release at 37 °C.

The hydrogels were prepared with FITC-labeled BSA, and the release studies were conducted at a physiological temperature (37 °C) and pH (PBS 7.4) in a bio-shaker at 200 rpm. The protein FITC-BSA was physically entrapped within the gel matrix during hydrogel formation. After gelation, the hydrogel surface was washed with water to remove any physisorbed protein. For monitoring protein release, hydrogels were placed in PBS and incubated at 37 °C with constant shaking (200 rpm). The release medium was replaced with fresh buffer at specific time points, and the amount of released FITC-BSA was deduced using UV–Vis spectroscopy. A slower release of protein was observed from the P2K hydrogel (58% at the end of 24 h). On the other hand, a considerably faster release of BSA molecules was achieved from the P8K hydrogels (Figure 5a). To confirm the on-demand release from the gel matrix, a protein-encapsulated hydrogel sample was subjected to passive diffusion in PBS (pH 7.4) for 4 h and then was treated with 10 mM DTT-containing PBS (pH 7.4). Upon addition of the reducing agent, the encapsulated cargo was rapidly released due to degradation of the gel matrix (Figure 5b).

### Cytotoxicity Evaluation

**Figure 6:**
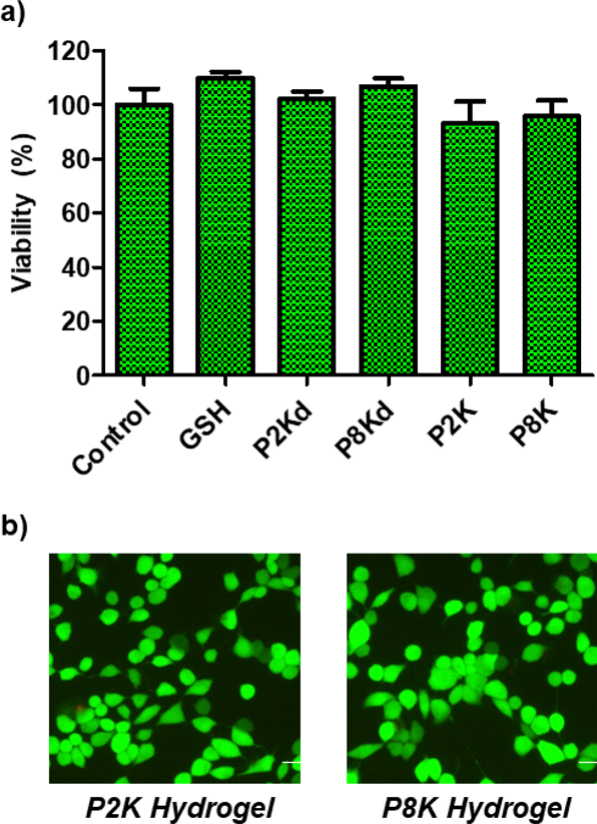
(a) Cell viability of L929 fibroblasts upon exposure to hydrogels fabricated with P2K and P8K and the degradation products of hydrogels P2K and P8K, denoted as P2Kd and P8Kd, respectively, and (b) fluorescence microscopy images of cells after live/dead staining.

The aim of designing the biodegradable, stimuli-responsive hydrogels in this work is to assess the potential of these scaffolds in employing them as biomaterials for delivery of therapeutic agents. Therefore, the evaluation of cytotoxicity of these hydrogels and their degradation products is important. The cytotoxicities of hydrogels and the degradation product obtained using exposure to the thiol-containing reducing agent (5 mM GSH) were investigated toward L929 mouse fibroblast cells. The CCK-8 viability assay on cells exposed to hydrogel samples (0.5 mg of P2K and P8K dry samples), their degradation products (P2Kd and P8Kd), and GSH (5 mM) revealed minimal toxicity and excellent cell viability (Figure 6a). This high level of biocompatibility of these hydrogels toward cells suggests their benign nature and thus makes them suitable for various biological applications. Additionally, a live/dead assay was performed on cells that were incubated with hydrogels. Incubated cells were stained with a mixture of calcein-AM and propidium iodide dyes, which indicate their live or dead state through green and red fluorescence, respectively. As can be seen from the predominance of green fluorescence in the cells (Figure 6b), one can conclude that the hydrogels were highly cytocompatible.

## Conclusions

A facile methodology for the fabrication of redox-responsive hydrogels is outlined. Readily accessible pyridyl-disulfide group containing telechelic PEG polymers upon mixing with tetra-thiol PEG polymers yields free standing hydrogels in high yields. Their facile formulation allows access to hydrogels under very mild and benign conditions, and thus provides a suitable strategy for encapsulation of delicate biomolecules of therapeutic interest. The release profile of the encapsulated protein can be tuned by appropriate choice of polymeric chain lengths. Importantly, obtained hydrogels undergo rapid degradation when treated with thiol-containing reducing agents, resulting immediate release of the encapsulated payload. One can envision that such on-demand degradable redox-responsive hydrogels will be of interest for various biomedical applications.

## Experimental Methods

### Materials

Poly(ethylene glycol)s (PEG, 2 kDa and 8 kDa), (5,5′-dithio-*bis*-(2-nitrobenzoic acid), fluorescein-5 maleimide, and 4-dimethylaminopyridine (DMAP) were purchased from Sigma Aldrich. Dithiothreitol (DTT) and 1-ethyl-3-(3-dimethylamino propyl) carbodiimide (EDC) were obtained from Alfa Aesar. Tetra-arm PEG_10K_-OH was purchased from Creative PEGWorks (USA). Anhydrous dichloromethane (CH_2_Cl_2_), tetrahydrofuran (THF), and toluene were obtained from SciMatCo Purification System. Other solvents and chemicals were purchased from Merck and were utilized as obtained. Ultrapure water was obtained by using a Milli-Q water purification System (Milli-Q system, Millipore, Billerica, MA, USA). PEG bis-acid,^36^ pyridyl disulfide-containing alcohol (PDS-OH),^37^ and tetra-arm PEG thiol^39^ were synthesized and characterized according to reported protocols.

### Measurements and Characterization

Polymer characterization was carried out with NMR spectroscopy (Varian 400 MHz). The microstructures of hydrogels were investigated with a scanning electron microscopy (SEM) instrument (JEOL NeoScope JCM-5000, an accelerating voltage of 10 kV). The rheological properties of gels were evaluated using a rheometer (Anton PAAR MCR 302). Encapsulation and release of fluorescently labeled model molecules were analyzed using a Varian Cary 50 Scan UV/vis spectrophotometer.

### Synthesis of Pyridyl Disulfide-Containing PEG (PEG-PDS)

Azeotropically dried bis-acid PEG_2K_ (0.45 mmol, 1 g), PDS-OH (1.36 mmol, 0.25 g), EDC (1.05 mmol, 0.202 g), and DMAP (0.091 mmol, 0.011 g) were dissolved in anhydrous CH_2_Cl_2_ (5 mL), and the reaction mixture was stirred at room temperature for 24 h under a nitrogen atmosphere. Further, the crude product was diluted with CH_2_Cl_2_ (30 mL) and extracted with saturated NaHCO_3_ solution (30 mL). The organic phase was collected and dried over anhydrous Na_2_SO_4_. The telechelic polymer PEG_2K_-PDS was obtained as a white powder after precipitation in cold diethyl ether (80% yield). ^1^H NMR (CDCl_3_, δ, ppm) 2.64 (m, 8H, OCC*H*_2_C*H_2_*OC), 3.03 (t, 4H, SC*H_2_*), 3.4–3.8 (m, 181H, OC*H_2_*C*H2*), 4.25 (t, 4H, C*H_2_*OCO), 4.34 (t, 4H, C*H_2_*OCO), 7.11 (d, 2H, NCC*H*), 7.58–7.68 (m, 4H, CHC*H*C*H*N), 8.45 (d, 2H, C*H*N). ^13^C NMR (CDCl_3_, δ, ppm) 172.2, 171.9, 149.8, 137.2, 120.9, 119.8, 69.1, 63.9, 62.5, 37.3, 28.9. The PEG_8K_-PDS polymer was synthesized according to the same protocol and was obtained with a yield of 82%. ^1^H NMR (CDCl_3_, δ, ppm) 2.64 (m, 8H, OCC*H*_2_C*H_2_*OC), 3.03 (t, 4H, SC*H_2_*), 3.4–3.8 (m, 727H, OC*H_2_*C*H2*), 4.25 (t, 4H, C*H_2_*OCO), 4.34 (t, 4H, C*H_2_*OCO), 7.11 (d, 2H, NCC*H*), 7.58–7.68 (m, 4H, CHC*H*C*H*N), 8.45 (d, 2H, C*H*N). ^13^C NMR (CDCl_3_, δ, ppm) 172.3, 171.9, 149.8, 137.1, 120.9, 119.8, 69.1, 63.9, 62.5, 37.3, 28.9.

### Preparation of Hydrogels

In a typical gelation procedure, the macromonomers PEG_2K_-PDS (10 mg, 3.8 mmol) and 4-arm PEG_10K_-SH (20.2 mg, 1.9 mmol) were dissolved separately in PBS solutions (pH 7.4, *V*_tot_ = 91.6 μL). The two solutions were then mixed and vortexed before placing in a shaker at 37 °C. Gelation occurred within few minutes, but the mixture was left for 24 h to ensure maximum chain end group coupling. The obtained hydrogel samples were rinsed with water to remove any unreacted polymers and byproducts and subsequently dried using lyophilization. The gelation yields of hydrogels were calculated gravimetrically. For loading of FITC-BSA, 50 μL of FITC-BSA solution (3 mg/mL) was mixed with PEG-PDS polymer solution, and then a solution of 4-arm PEG_10K_-SH in PBS was added to it, and the mixture was vortexed to ensure thorough mixing before gelation.

### Swelling of Hydrogels

Freshly prepared hydrogel samples were freeze-dried (5 mg) and immersed in deionized water (5 mL) at room temperature. At periodic time intervals, the increase in the mass of hydrogels was recorded after removing the surface absorbed water by the help of a moisturized filter paper. The swelling percentage was calculated as (*M*_s_ – *M*_d_)/*M*_d_ × 100, where *M*_s_ and *M*_d_ are the weights of swollen and dry hydrogels, respectively. Swelling experiments were repeated for three different samples, and an average was plotted with standard deviation.

### End-Group Consumption Determination

The freshly prepared dry hydrogel sample (5 mg) was transferred into a DTT solution (3 mL, 10 mM in PBS) in a vial and incubated at 37 °C. The absorbance at 343 nm specific to 2-mercaptopyridine released upon disulfide bond cleavage was then measured by UV–Vis spectroscopy. The total amount of 2-mercaptopyridine was calculated by the molar extinction coefficient of 2-mercaptopyridine,^37^ and the percent release was obtained by the following equation: *n*_rel_. (%) = (*n*_act_/*n*_theo_.) × 100.

### Thiol-Content Determination

The residual thiol content was investigated by Ellman’s test according to the following procedure after gelation. A reaction medium of PBS (pH 8.0) containing 1 mM EDTA buffer was prepared. Ellman’s reagent solution was prepared by dissolving 4 mg of Ellman’s reagent (5, 5-dithio-bis-(2-nitrobenzoic acid)) in 1 mL of previously prepared PBS (pH 8.0) solution. A freshly prepared dry hydrogel sample (5 mg) was placed in a vial with 2.5 mL of PBS, and 1 mL of Ellman’s reagent solution was added on it. The mixture was incubated at 37 °C for 2 h. Further, the absorbance at 412 nm was measured by UV–Vis spectroscopy to calculate the total sulfhydryl group content in the hydrogel sample according to the Beer’s law using the molar extinction coefficient of TNB^2–^ (14,150 M^–1^ cm^–1^).^44^

### Morphological Analysis

Surface morphologies of lyophilized hydrogel samples were analyzed using a scanning electron microscope (SEM), an ESEM Philips XL-30 (Philips, Eindhoven, and The Netherlands) instrument, operating at an accelerating voltage of 10 kV.

### Rheological Measurements

Rheological behaviors of hydrogels were evaluated by using an Anton Paar MCR 302 rheometer. A circular plate (15 mm diameter) was used as test geometry. Time sweep and frequency sweep tests (0.1–100 Hz; 1% strain) were applied. A closed system was used to avoid evaporation of water from hydrogels during measurements. Furthermore, for self-healing tests, the hydrogels were subjected to multiple cycles of high strain (600%) (120 s) and low strain (1%) (100 s) under constant frequency (1 Hz) and changes in storage and loss modulus were measured.

### Self-Healing Studies

The 33% (w/v) hydrogels (with and without a red food dye) were prepared in a vial. The wet gels were cut into two semicircular pieces using a surgical blade. One of the halves of the colored piece and the other piece (without dye) were pressed against each other in the same orientation as the cut. After 5 min in contact, the self-healed hydrogel was stretched into opposite directions to confirm robust sticking of the two halves. For rheological assessment of self-healing, the freshly prepared P8K hydrogel was swollen into equilibrium and subjected to high (600%) and low (1%) strains in cycles.

### Redox-Responsive Degradation

Visual and rheological experiments were carried out to demonstrate the disintegration of the gel matrix in the presence of 10 mM DTT at 37 °C. Degradation of the gel was investigated using time sweep tests on a rheometer. For visual assessment of degradation, fluorescein-maleimide-conjugated hydrogels were used. To conjugate a fluorescent label, a pre-weighed piece of hydrogel was placed in a glass vial in DMSO (3 mL) and fluorescein-5 maleimide corresponding to the amount of free thiol groups (as determined by Ellman analysis) was added onto the gel. After 2 h, the gel was sequentially washed with excess DMSO and water. The sample was divided into two pieces. One of the gel pieces was placed in a vial containing 0.5 mL of DTT (10 mM) and the other piece was immersed in PBS solution. Photographs of vials were taken under UV illumination (365 nm) to indicate the gel degradation and distinct color changes of the immersion medium.

### Protein Loading and Release Studies

Fluorescein isothiocyanate-labeled bovine serum albumin (FITC-BSA) was encapsulated into hydrogels during gelation as described above. After gelation, hydrogels were placed into a PBS (pH 7.4, 1 mL)-containing vial and incubated at 37 °C with constant shaking (200 rpm). Release media were periodically replaced with fresh solutions, and the amount of BSA released in the supernatant was determined using a UV–vis spectrophotometer at 495 nm.^45^ For the on-demand release study, hydrogels were treated with 10 mM DTT after 4 h of passive release. Three different release experiments were performed for each set of hydrogels.

### *In Vitro* Cytotoxicity Analysis

The cytotoxic activity of hydrogels was investigated on L929 mouse fibroblast cells cultured in RPMI 1640 media supplemented with fetal bovine serum (FBS, 10%), l-glutamine, and penicillin/streptomycin. 6000 cells/well were seeded in a 96-well plate as quadruplicates and incubated at 37 °C for 12 h. Cells were treated with gels (0.5 mg/well) at 37 °C for 48 h. After incubation hydrogels were removed, and cells were washed with PBS (100 μL/well) twice. The CCK-8 assay was performed to determine cell viability. Briefly, cells were incubated with 10% CCK-8 solution (100 μL/well) for 1 h and absorbance at 450 nm was measured using a microplate reader. GraphPad Prism software was used for viability calculations. Also, 10 mg of each gel (P2K and P8K) was degraded in 5 mM glutathione (3 mL). Thereafter, L929 fibroblast cells were treated with degradation products (1:10 final dilution in RPMI) followed by viability measurements.

### Live/Dead Cell Viability Assay

L929 cells were seeded into a 12-well plate (250,000 cells/well) with DMEM low glucose media and incubated at 37 °C overnight to grow and adhere. After the incubation step, cells were washed three times with PBS and treated with P2K and P8K hydrogels (1 mg) for 24 h. After 24 h, hydrogels were removed, and cells were washed three times with PBS. The cells were stained according to a live/dead assay kit protocol (Sigma, 04511-1KT-F). First, the cells were stained in 0.5 mL of the PBS (5 mL) supplemented with 10 μL of calcein-AM (stained live cells) and 5 μL of propidium iodide (PI; stained dead cells) in PBS solution for 30 min at 37 °C. After removing the solutions and washing with PBS, the stained cells were observed by using a fluorescence microscope (Zeiss Observer A1 equipped with AxioCam MRc5) and processed with AxioVision software. Green fluorescence from calcein-AM staining indicates the presence of live cells, and the red fluorescence of cells exposed to propidium iodide suggests their dead state.

## Supporting Information Available

^1^H NMR, ^13^C NMR, and FTIR spectra and GPC chromatograms of hydrogel precursors; rheological plots and GSH-induced degradation photographs of hydrogels (PDF)
