# Supplementary material for: Redox-Responsive Hydrogels for Tunable and “On-Demand” Release of Biomacromolecules
Source: Bioconjug Chem. 2022 Apr 21;33(5):839–47. doi: 10.1021/acs.bioconjchem.2c00094 (PMC9121344; doi:10.1021/acs.bioconjchem.2c00094)
Supplement: Supplementary file 1 — bc2c00094_si_001.pdf [file bc2c00094_si_001.pdf]

Supporting Information

Redox-Responsive Hydrogels for Tunable and 'On-Demand' Release of Biomacromolecules

Ruveyda Kilic Boz,[a, †] Duygu Aydin,[a, †] Salli Kocak,[a] Bianka Golba,[a]

Rana Sanyal,[a,b] and Amitav Sanyal *,[a,b]

[a] *Department of Chemistry, Bogazici University, Istanbul 34342, Turkey*
[b] *Center for Life Sciences and Technologies, Bogazici University, Istanbul, Turkey*

† These authors contributed equally to this study.

* Corresponding authors: Email address: amitav.sanyal@boun.edu.tr.

LIST OF FIGURES

Figure S1. ^{1}HNMR spectrum of P2K-COOH in $CDCl_3$... 3

Figure S2. ^{1}HNMR spectrum of P8K-COOH in $CDCl_3$... 3

Figure S3. ^{1}HNMR spectrum of P2K-PDS in $CDCl_3$. 4

Figure S4. ^{1}HNMR spectrum of P8K-PDS in $CDCl_3$. 4

Figure S5. ^{13}CNMR spectrum of P2K-PDS in $CDCl_3$. 5

Figure S6. ^{13}CNMR spectrum of P8K-PDS in $CDCl_3$. 5

Figure S7. GPC chromatograms of linear PEG-OH (left) and PDS-functionalized PEG (right). 6

Figure S8. FTIR spectra of P2K-OH (A), P2K-PDS (B), P8K-OH (C), P8K-PDS (D). 6

Figure S9. Strain dependent deformation of hydrogel (P8K)... 7

Figure S10. Rheological self-healing test for P8K hydrogel (40 mg) soaked in ethyl-maleimide containing PBS solution (0.6 mg/mL) showing storage modulus (G′) and loss modulus (G′′) at alternating strains of 1 and 600% (three cycles). 7

Figure S11. Visual degradation of dye-conjugated hydrogel (P8K) in GSH (5 mM) and PBS at 37 °C.....7

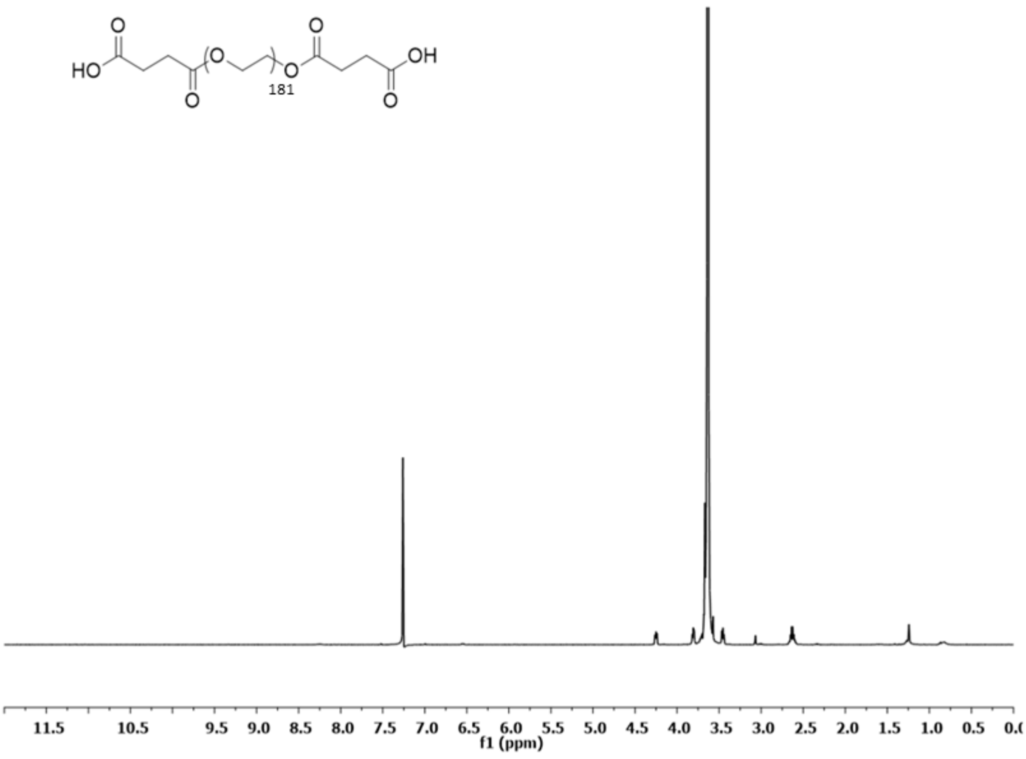

Figure S1. ^{1}HNMR spectrum of P2K-COOH in $CDCl_3$.

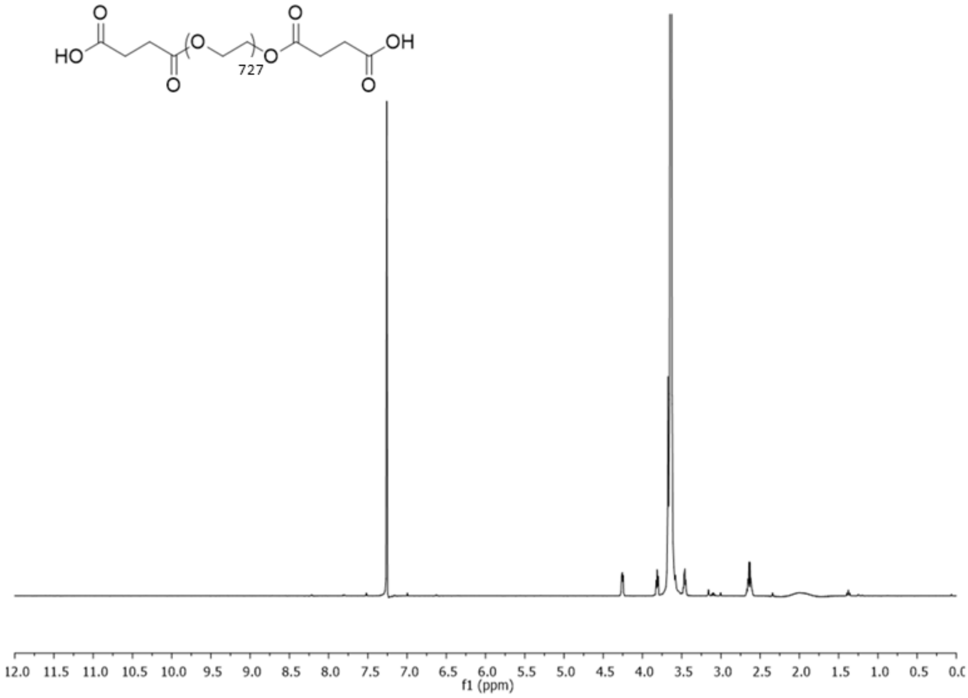

Figure S2. ^{1}HNMR spectrum of P8K-COOH in $CDCl_3$.

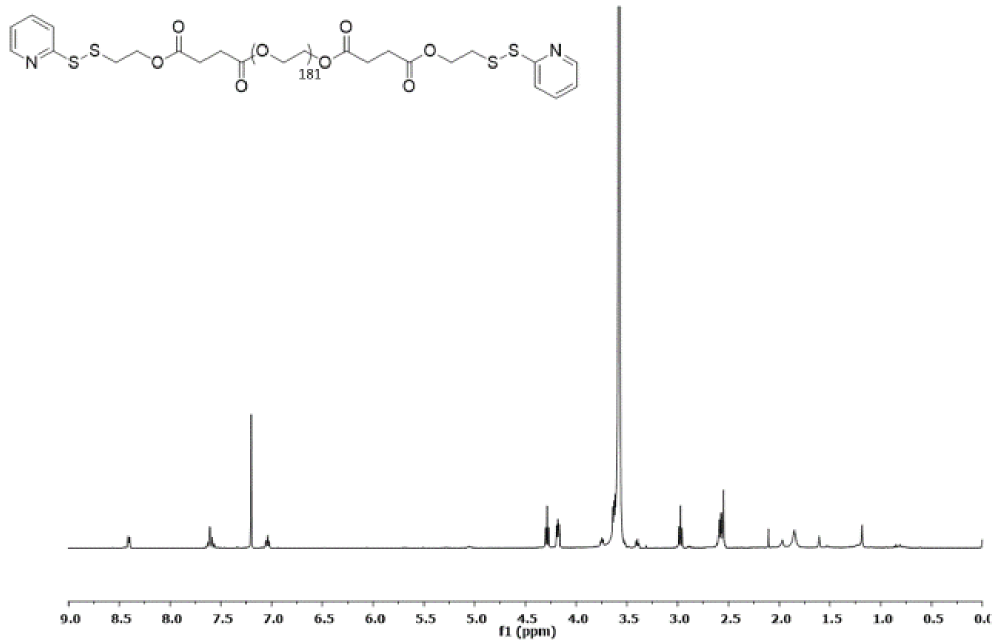

Figure S3. ^{1}HNMR spectrum of P2K-PDS in $CDCl_3$.

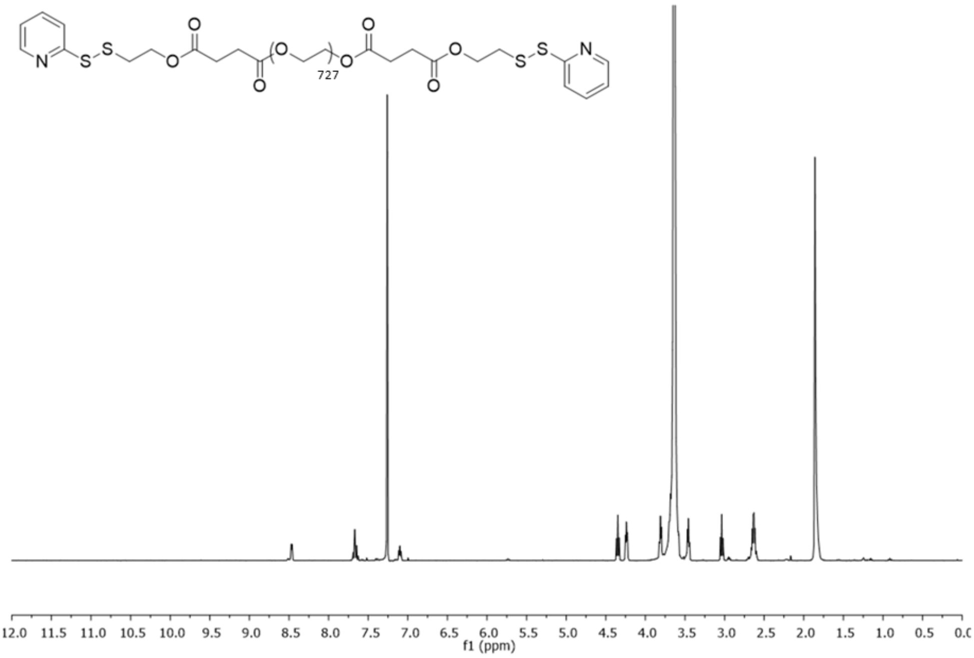

Figure S4. ^{1}HNMR spectrum of P8K-PDS in $CDCl_3$.

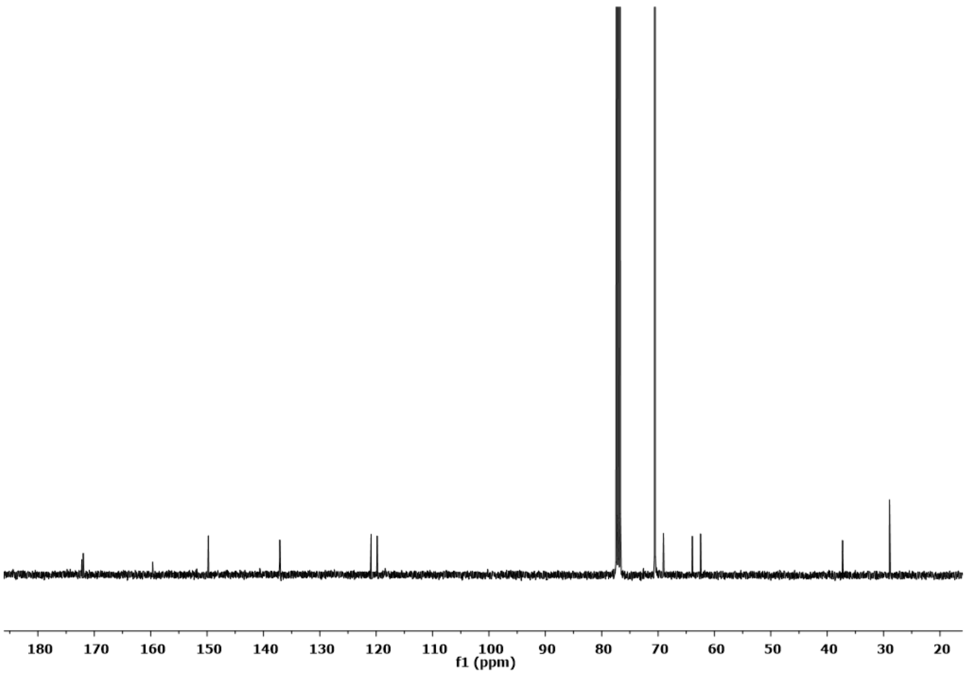

Figure S5. ^{13}CNMR spectrum of P2K-PDS in $CDCl_3$.

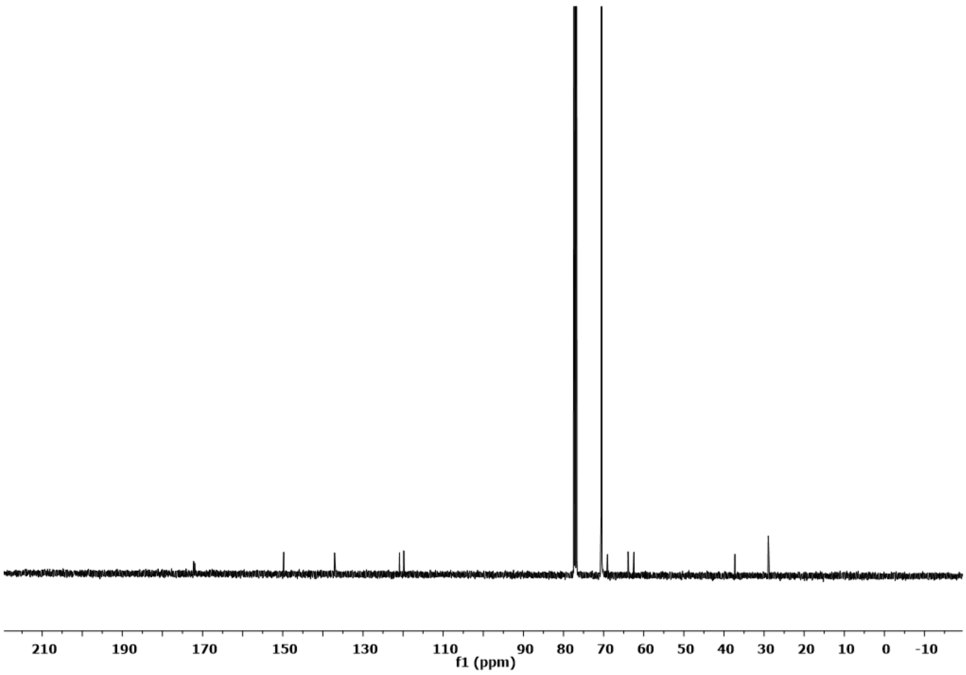

Figure S6. ^{13}CNMR spectrum of P8K-PDS in $CDCl_3$.

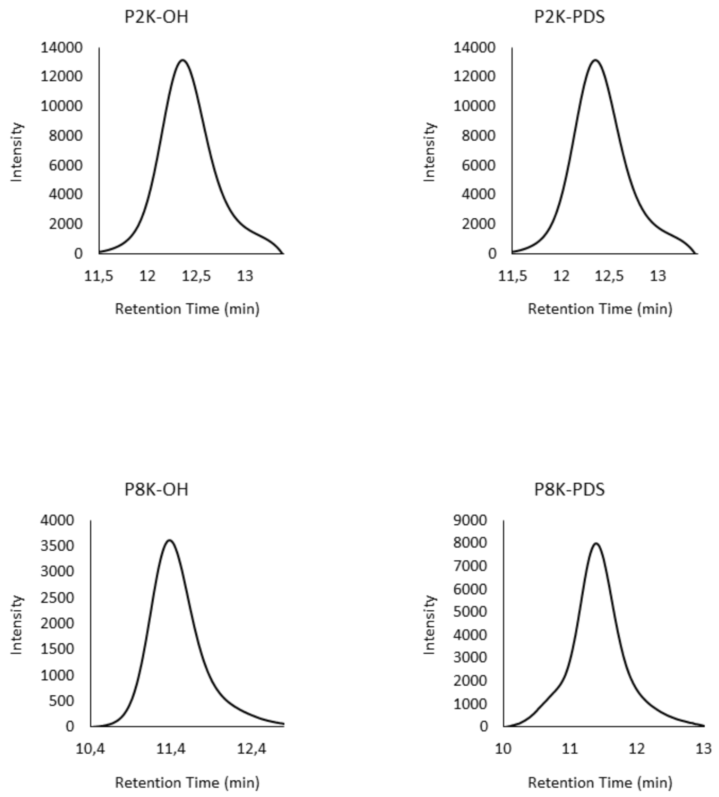

Figure S7. GPC chromatograms of linear PEG-OH (left) and PDS-functionalized PEG (right).

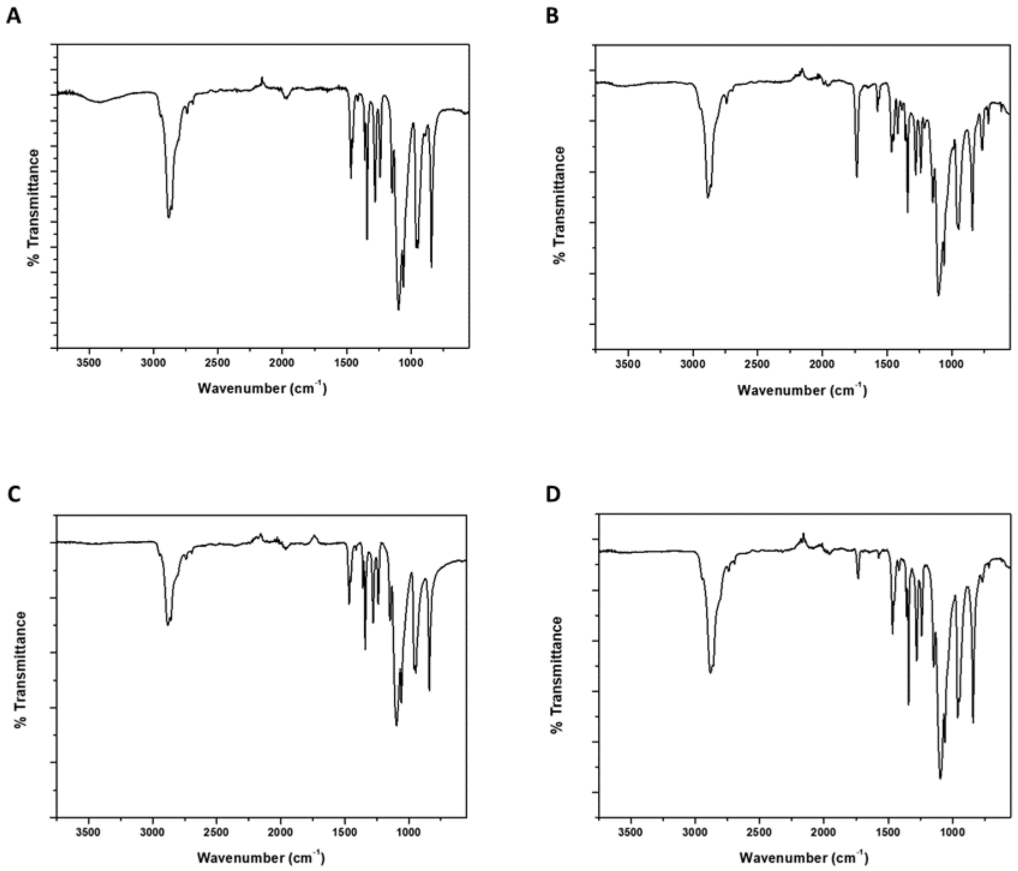

Figure S8. FTIR spectra of P2K-OH (A), P2K-PDS (B), P8K-OH (C), P8K-PDS (D).

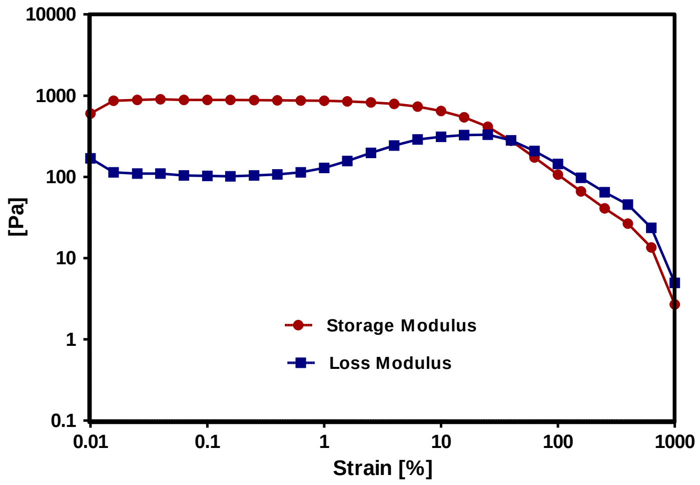

Figure S9. Strain dependent deformation of hydrogel (P8K).

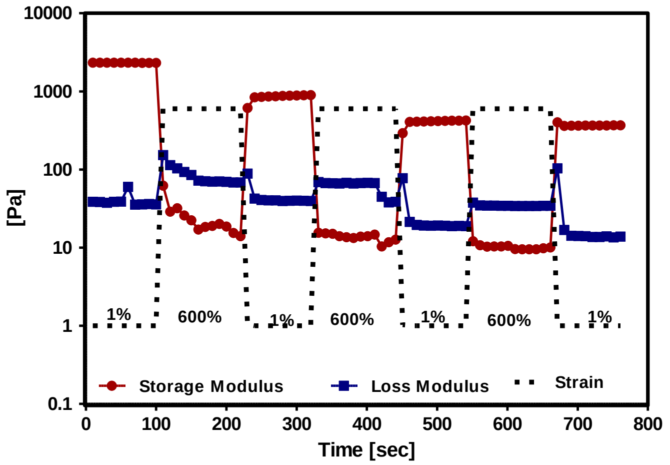

Figure S10. Rheological self-healing test for P8K hydrogel (40 mg) soaked in ethyl-maleimide containing PBS solution (0.6 mg/mL) showing storage modulus (G') and loss modulus (G'') at alternating strains of 1 and 600% (three cycles).

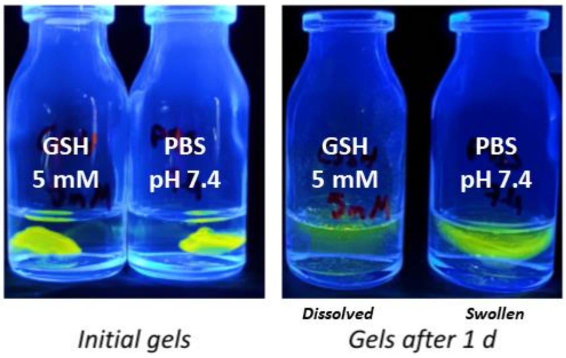

Figure S11. Visual degradation of dye-conjugated hydrogel (P8K) in GSH (5 mM) and PBS at 37 °C.
